# The first mitochondrial genome of spongillafly from Asia (Neuroptera: Sisyridae: *Sisyra aurorae* Navás, 1933) and phylogenetic implications of Osmyloidea

**DOI:** 10.1080/23802359.2021.1951142

**Published:** 2021-07-19

**Authors:** Xiumei Lu, Aili Lin, Dongsheng Wang, Xingyue Liu

**Affiliations:** a Institute of Ecological and Environmental Protection, Shanghai Academy of Agricultural Sciences, Shanghai, China; b Department of Entomology, China Agricultural University, Beijing, China

**Keywords:** Mitogenome, Sisyridae, Osmyloidea, phylogeny

## Abstract

The spongillafly species *Sisyra aurorae* Navás, 1933 (Neuroptera: Sisyridae) is an endemic species in China and is first recorded from Shanghai. The mitogenome of this species is sequenced, representing the first mitogenome of Sisyridae from Asia. The nearly complete mitogenome is 15,634 bp, which contains 13 protein-coding genes (PCGs), 22 transfer RNA genes (tRNAs), two ribosomal RNA genes (rRNAs), and a control region. The gene order and arrangement are similar to other lacewing mitogenomes. Both Bayesian and maximum likelihood analyses based on 13 PCGs recovered the interfamilial phylogeny within Osmyloidea as Sisyridae + (Nevrorthidae + Osmylidae).

Sisyridae (spongillaflies) is a small lacewing family (Neuroptera: Osmyloidea), comprising about 70 species in four genera worldwide (Oswald 2020). The larvae are exclusively aquatic as a specialist predator of freshwater sponges and can be used as indicators of freshwater quality (Parfin and Gurney 1956; Weissmair 1994, 2005). Hitherto, only two partial mitochondrial genomes have been determined for the sisyrids respectively from Europe and North America, namely *Sisyra nigra* (Retzius 1783) and *Climacia areolaris* Hagen, 1861 (Wang et al. 2017). Currently, only six sisyrid species in two genera are recorded from China (Oswald 2020). Here, the mitochondrial genome of the Chinese endemic species *Sisyra aurorae* Navás, 1933 is sequenced, which represents the first mitogenome of Sisyridae from Asia.

The specimen was collected from Zhuanghang town, Fengxian District, Shanghai, China (121.39174°E, 30.89438°N), representing the first record of Sisyridae from Shanghai. It is deposited at the Pest Control Lab of Shanghai Academy of Agricultural Sciences under the voucher number SAAS20190624 (contact: Xiumei Lu, lxm2361892563@126.com). The genomic DNA was extracted from tissues of the thorax and legs using Hipure Universal DNA Kit, Magen. The mitogenome was sequenced by Illumina NovaSeq with 250 bp paired-end reads, assembled with A5-miseq and SPAdes, and annotated with MITOs WebServer. The phylogenetic tree is conducted under CIPRES and IQtree WebServers.

The mitochondrial genome of *S. aurorae* is a typical circular DNA with 15,634 bp in length, which consists of a set of 37 genes (13 PCGs, 22 tRNAs, and two rRNAs) and a control region. The length is longer than previous two sisyrid mitogenomes, while the 16S rRNA and control region are not completely sequenced here. The gene order is identical to that of the putative ancestral arrangement of insects. The H chain codes 23 genes, while the other 14 genes are coded by the L chain. The nucleotide composition of the mitogenome is biased toward A and T, with 79.8% of A + T content (A = 40.6%, T = 39.2%, C = 11.6%, G = 8.5%). There are 616 bp intergenic nucleotides in nine locations, ranging from 1 to 586 bp in length, while only 51 bp overlapped nucleotides in 15 locations, ranging from 1 to 9 bp in length. All PCGs are started with ATN (ATG in *cox2*, *atp6*, *cox3*, *nd4*, *nd4l*, *cytb*; ATT in *nd2*, *cox1*, *atp8*, *nd5*, *nd6*; ATA in *nd3*) except *nd1* started with TTG. The typical stop codon TAA is adopted by eight PCGs (*nd2*, *cox1*, *atp8*, *atp6*, *cox3*, *nd4l*, *nd6*, *cytb*), and TAG occurs in two PCGs (*nd3* and *nd1*); the stop codon remains unknown in three PCGs (*cox2*, *nd5* and *nd4*). The 22 tRNA genes range from 63 to 72 bp in length, and all could be folded into canonical cloverleaf structure. Notably, the size of TΨC loop varies and is truncated in *trnI*, *trnF* and *trnL1*.

The superfamily Osmyloidea includes three families, i.e., Sisyridae, Nevrorthidae and Osmylidae. The larvae of these families are aquatic or semi-aquatic, while the adults are terrestrial. Though recent molecular studies support the close affinities among these three families, the phylogenetic relatinship among them are still elusive with inconsistent results recovered from different data (Aspöck and Aspöck 2008; Winterton et al. 2010, 2018; Wang et al. 2017; Vasilikopoulos et al. 2020). Here, the phylogenetic relationship of Osmyloidea were inferred based on 13 PCGs from 14 species using Bayesian and maximum likelihood methods (Figure 1). The monophyly of these families are strongly supported. The result shows that Sisyridae is sister to the clade including Nevrorthidae and Osmylidae, being consistent with that previously recovered in Winterton et al. (2018).

**Figure 1.**
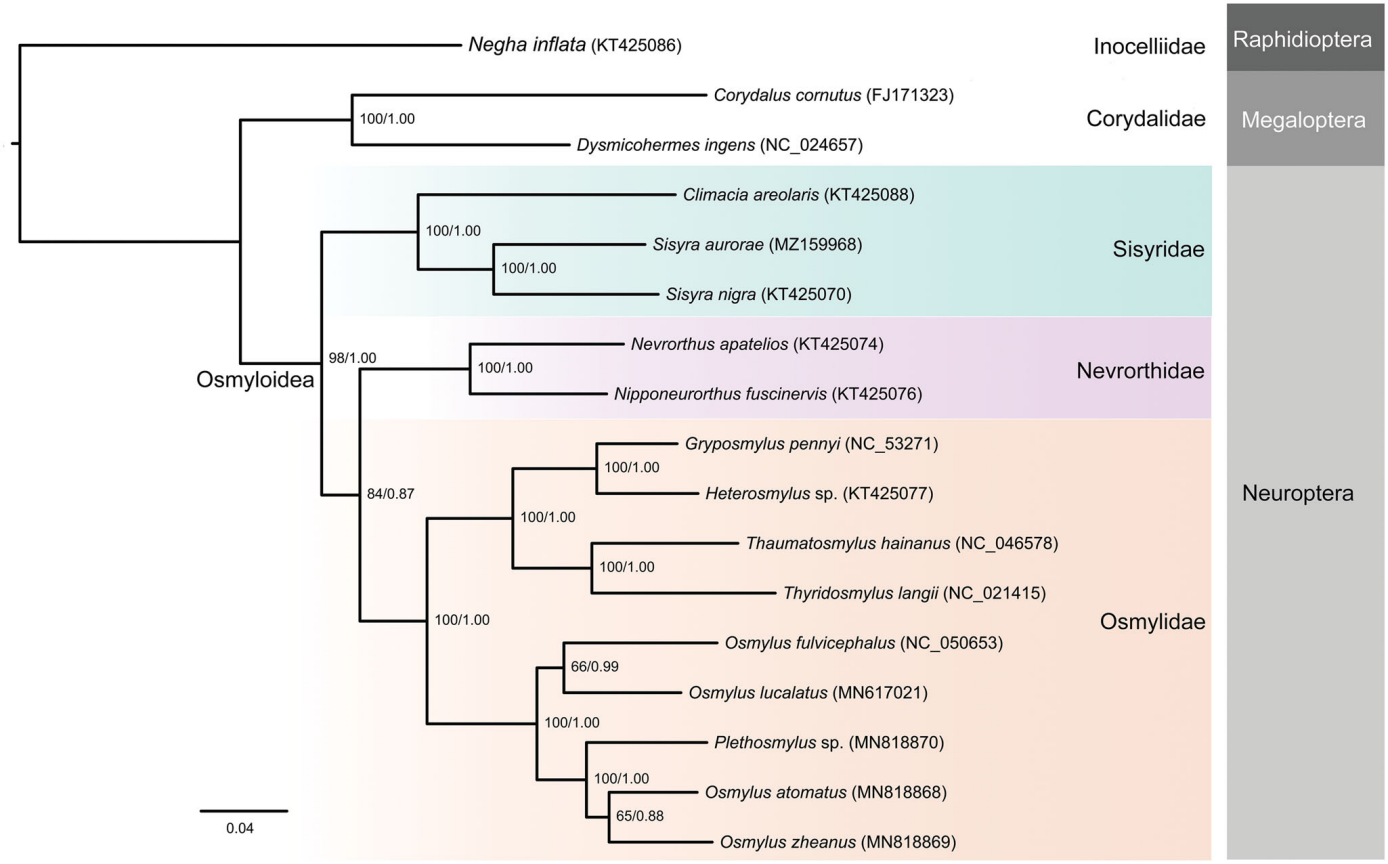
Phylogenetic relationship of Osmyloidea inferred based on 13 PCGs using Bayesian and maximum likelihood (ML) methods. The ML bootstrap support values and posterior probabilities are shown above each. Genbank accession numbers for the sequences are indicated next to the species names.

## Nucleotide sequence accession number

The mitogenome sequence that supports the findings of this study is openly available in GenBank of NCBI at (https://www.ncbi.nlm.nih.gov/nuccore/MZ159968) under the accession number MZ159968. The associated BioProject, BioSample and sequence read archive (SRA) numbers are PRJNA729218, SAMN19114475 and SRR14508920 respectively.
